# Dysfunctional Immune Response in Acute-on-Chronic Liver Failure: It Takes Two to Tango

**DOI:** 10.3389/fimmu.2019.00973

**Published:** 2019-05-01

**Authors:** Rosa Martin-Mateos, Melchor Alvarez-Mon, Agustín Albillos

**Affiliations:** ^1^Department of Gastroenterology and Hepatology, Centro de Investigación Biomédica en Red de Enfermedades Hepáticas y Digestivas (CIBERehd), Hospital Universitario Ramón y Cajal, Instituto Ramón y Cajal de Investigación Sanitaria (IRYCIS), Instituto de Salud Carlos III, Universidad de Alcalá, Madrid, Spain; ^2^Department of Immune System Diseases and Oncology, Centro de Investigación Biomédica en Red de Enfermedades Hepáticas y Digestivas (CIBERehd), Hospital Universitario Príncipe de Asturias, Instituto Ramón y Cajal de Investigación Sanitaria (IRYCIS), Instituto de Salud Carlos III, Universidad de Alcalá, Madrid, Spain

**Keywords:** ACFL, systemic inflammation, cirrhosis-associated immune dysfunction, immune paralysis, liver, cirrhosis

## Abstract

Acute-on-chronic liver failure (ACLF) is characterized by the acute decompensation of cirrhosis associated with organ failure and high short-term mortality. The key event in the pathogenesis is a dysfunctional immune response arising from exacerbation of the two main immunological alterations already present in cirrhosis: **systemic inflammation** and **immune cell paralysis**. High-grade systemic inflammation due to predominant activation and dysregulation of the innate immune response leads to the massive release of cytokines. Recognition of acutely increased pathogen and damage-associated molecular patterns by specific receptors underlies its pathogenesis and contributes to tissue damage and organ failure. In addition, an inappropriate compensatory anti-inflammatory response over the course of ACLF, along with the exhaustion and dysfunction of both the innate and adaptive immune systems, leads to functional immune cell paralysis. This entails a high risk of infection and contributes to a poor prognosis. Therapeutic approaches seeking to counteract the immune alterations present in ACLF are currently under investigation.

## Introduction

Cirrhosis is characterized by progressive fibrosis, portal hypertension, and liver failure. It comprises two consecutive but potentially reversible stages: compensated and decompensated cirrhosis. Cirrhosis also features a progressively dysfunctional immune response that encompasses systemic inflammation (SI) and immunodeficiency. The term cirrhosis-associated immune dysfunction (CAID) (1) defines the spectrum of immune system alterations present in cirrhosis, which induce extrahepatic clinical manifestations and a higher susceptibility to bacterial infection.

Over the course of compensated or decompensated cirrhosis, an acute precipitating event, such as bacterial infection, may challenge liver homeostasis producing a syndrome called acute-on-chronic liver failure (ACLF). ACLF is characterized by acute decompensation of cirrhosis, hepatic and/or extrahepatic organ failure and high short-term mortality (2). The activation of an inadequate immune response to the triggering event is key to its pathogenesis and involves the highest grade of SI and immunodeficiency. This review is an overview of the major immune alterations that occur in ACLF, considering the specific circumstances of patients with end-stage liver disease.

## ACLF: Concept and Definition

The most widely accepted definition of ACLF arises from the results of a large, prospective, multicenter European project, the CANONIC study (2). According to its results, ACLF is defined in terms of its three main features:

1. *Acute decompensation* of cirrhosis, which refers to the acute development or significant worsening of ascites, encephalopathy, gastro-intestinal bleeding or any combination of these in a patient with cirrhosis. ACLF may occur at any stage from compensated to decompensated cirrhosis, and can be triggered by hepatic or extrahepatic factors (2).
2. *Organ failure*, including both the liver and extrahepatic organs. Organ failure is the diagnostic hallmark of ACLF. The kidney is the most commonly failing organ (55.8%) followed by the liver (43.6%) (2). According to the affected organ and number of organs failing, ACLF severity is graded as three stages (ACLF 1-3).
3. *High short-term mortality*, 28-day mortality is poor and correlates with severity, ranging from 23 to 74% in patients with ACLF grades 1 to 3, respectively (2).

## Immune Dysfunction in ACLF

**Figure 1:**
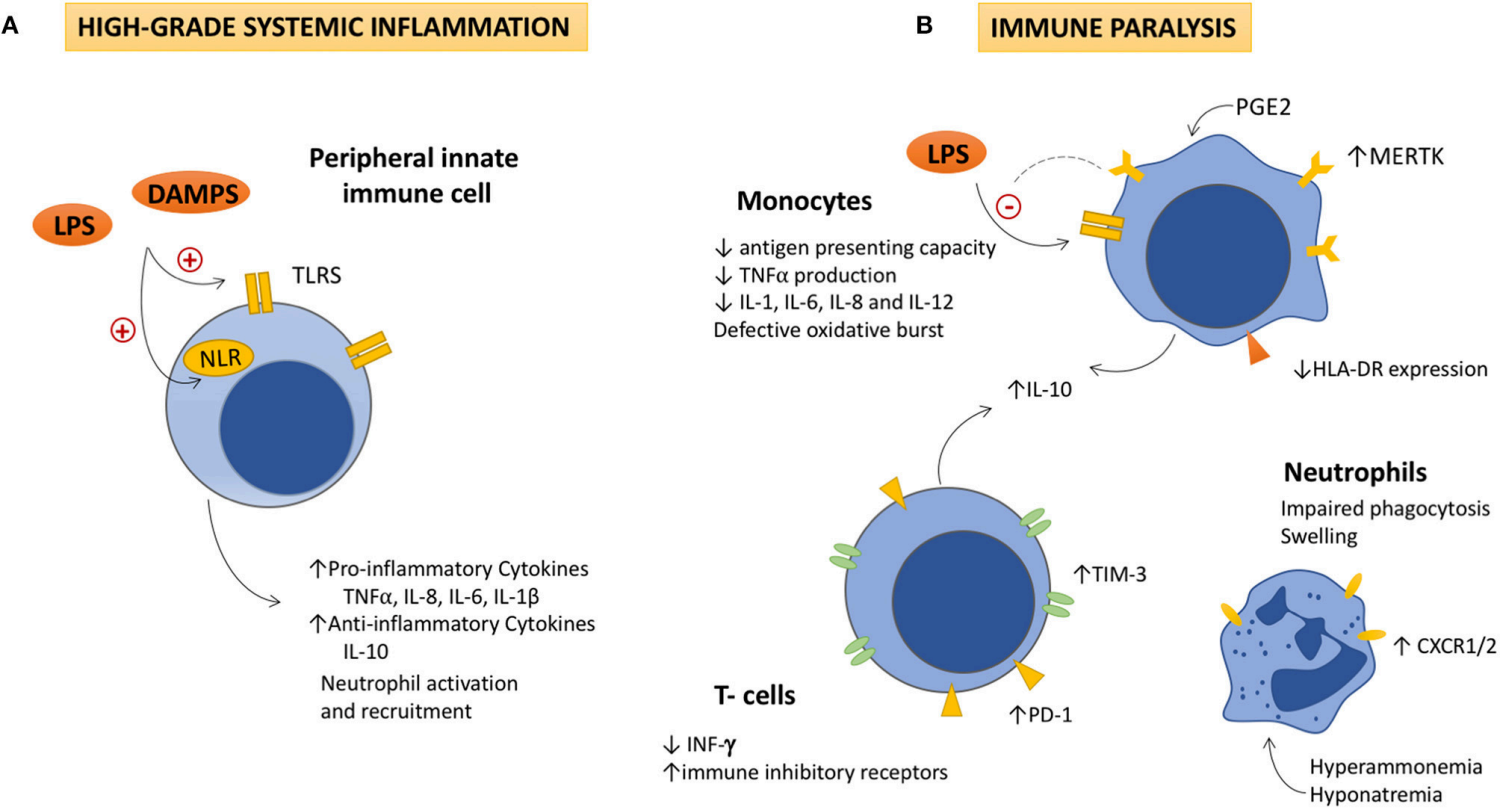
The dysfunctional immune response in ACLF is featured by high-grade systemic inflammation and immune cell paralysis. **(A)** High-grade systemic inflammation. Activation of Toll like and Nod-like receptors in peripheral monocytes and dendritic cells by increased DAMPs and PAMPs, leads to the overproduction of pro-inflammatory and anti-inflammatory cytokines, as well as to the activation and recruitment of neutrophils to the site of injury. Detrimental effects due to the excessive immune cell activation (immunopathology), contribute to tissue damage, and organ failure, which also generates more DAMPs and perpetuates immune activation. **(B)** Immune cell paralysis. As ACLF progresses, (i) an excessive compensatory anti-inflammatory response, (ii) the exhaustion of the immune effectors, and (iii) metabolic and neuroendocrine disturbances (increased PGE2, hyperammonemia, and hyponatremia), lead to an acquired immunodeficiency that involves the innate and the adaptive responses. IL-10 seems to play a pivotal role in the pathogenesis of the immune cell paralysis as it modulates NF-kβ activity decreasing TNFα, IL-1, IL-6, IL-8, and IL-12 secretion by monocytes. IL-10 inversely correlates with levels of immune inhibitory receptors in monocytes (MERTK), lymphocytes (PD-1 and TIM-3), and INF-γ production, which contributes to the immune cell dysfunction. Decreased HLA-DR expression in monocytes impairs their antigen presenting role and TNFα production in response to LPS stimulation. Additionally, neutrophils show high levels of CXCR1/CXCR2 receptors, which contributes to hepatocyte death through early apoptosis and necrosis. LPS, Lipopolysaccharides; DAMPs, damage associated molecular patterns; TLR, Toll like receptors; NRL, nod like receptors; TNFα: Tumor necrosis factor α; IL, interleukin; PGE2, Prostaglandin E2; MERTK, tyrosine-protein kinase MER; IFN-γ, interferon-γ; TIM-3, T-cell immunoglobulin and mucin domain 3; PD-1, Programmed cell death-1; CXCR1/2, chemokine receptor1/2.

The key immune pathogenic events in ACLF are exacerbated SI and worsening of the defective effector immune response already present in cirrhosis (Figure 1). Both are general mechanisms leading to organ failure in other clinical scenarios, such as sepsis, in otherwise immunocompetent subjects (3). In cirrhosis, however, systemic inflammation and immune cell anergy give rise to more severe consequences and a worse prognosis due to preexisting immune dysfunction and liver damage.

### Cirrhosis-Associated Immune Dysfunction

Cirrhosis, independently of stage and etiology, is associated with a spectrum of distinctive abnormalities of the immune system that result in SI and acquired immunodeficiency, collectively termed CAID (1). The severity of CAID parallels the cirrhosis stage, and can worsen acutely as the consequence of incidental events, such as bacterial infection or hepatic inflammation (i.e., alcoholic hepatitis). The immune system phenotype of patients with decompensated cirrhosis and, to a lesser extent, those with compensated disease, features low-grade systemic inflammation associated with the increased expression of activation antigens and production of pro-inflammatory cytokines by the activated circulating and tissue-resident immune cells. At this stage, the effector immune response against pathogens is not yet fully compromised. Further, the most severe immune disturbances in cirrhosis are found in patients with ACLF who present an immune system phenotype characterized by high-grade systemic inflammation and severe immunodeficiency. CAID exemplifies the dynamic nature and plasticity of the immune response, as it can switch from a predominantly low-grade pro-inflammatory phenotype to another response involving massive systemic inflammation and marked immune cell anergy, and vice versa.

### Systemic Inflammation in ACLF

#### Evidence of SI in ACLF

Inflammation is triggered by various noxa as a host response designed to restore tissue homeostasis. In cirrhosis, the greatest inflammatory responses are those associated with bacterial challenge or massive hepatocyte damage targeted at eliminating bacteria and promoting tissue repair. Eventually, immune activation becomes massive and results in an overwhelming production of pro-inflammatory cytokines, which leads to tissue damage and organ failure. Evidence of high-grade SI in ACLF is supported by increased plasma levels of pro-inflammatory (IL-6 and TNFα) and anti-inflammatory cytokines (IL-10 and IL-1ra) (4), soluble markers of macrophage activation (sCD163 and mannose receptor) (5), C-reactive protein, and circulating numbers of white blood cells (2). These biomarkers are moderately increased in patients with acutely decompensated cirrhosis without organ failure, but markedly augmented in those with ACLF, which indicates more severe inflammation in the latter, similar to the degree observed in patients with sepsis admitted to intensive care units (6, 7). Such increased levels of inflammatory markers closely correlates with the number of failing organs, and therefore, with severity and prognosis (8). Additionally, the best characterized triggers of ACLF (bacterial infection and alcoholic hepatitis) are recognized inductors of the inflammatory response, and SI is even present when no identifiable precipitating factors are found at presentation. Together these findings suggest that inflammation plays a major pathogenic role in ACLF.

#### Mechanisms of Systemic Inflammation

A. **General mechanisms of SI in cirrhosis**

Infection and tissue injury are the classically known inducers of inflammation. Bacterial pathogens of a given class present sets of conserved molecular patterns (PAMPs) that can be recognized by pattern recognition receptors (PRRs), such as Toll- (TLRs) and NOD-like receptors (NLRs) (9). Recognition of PAMPs by PRRs activates intracellular signaling pathways leading to cytokine production and activation and recruitment of neutrophils. However, inflammation in the absence of pathogens (sterile inflammation) also occurs, as PRRs may recognize molecules released by dead or injured cells. These molecules (damage-associated molecular patterns or DAMPs) are intracellular factors usually invisible to the host immune system, thus preventing pathological inflammation and autoimmunity (10).

In cirrhosis, low-grade systemic inflammation is prompted by increased intestinal barrier permeability due to loosening of the tight-junctions, higher bacterial transcytosis, and a reduction in the mediators that limit contact between bacteria and intestinal microvilli. In addition, bacterial overgrowth and dysbiosis promote translocation of bacteria and PAMPs, such as LPS, from the intestinal lumen to the mesenteric lymph nodes and gut-associated lymphoid tissue. The capacity of the lymph nodes to eliminate intestinal bacteria may be eventually overcome, allowing gut-derived PAMPs to spread, reach the liver, and activate proinflammatory signaling pathways. Moreover, DAMPS released from chronically injured or dead hepatocytes, such as high mobility group box 1, are also recognized by PRRs contributing to persistent SI in cirrhosis (11). The analysis of the microbiota in portal vein as in other different locations (liver outflow, central venous blood, and peripheral venous blood), has recently shown that the microbial composition does not differ significantly among the circulatory compartments. Cytokine levels in serum directly correlated with the abundance of blood microbiome genera, providing further evidence of the association between circulating microbiome and inflammation in cirrhosis (12).

B. **Specific mechanisms active in ACLF**

ACLF is characterized by an extreme exacerbation of the low-grade SI already present in cirrhosis. Cytokines involved in the innate immune response are significantly up-regulated, suggesting the predominant pathogenic involvement of the innate arm of the immune system. In addition, different cytokine profiles according to the precipitating event have been observed: thus, IL-8 is significantly higher in alcoholic patients, while TNFα, IL-6, and IL-1ra are mostly increased when bacterial infection is the trigger (4).

##### Sepsis-induced ACLF

Bacterial infection is the most frequent identifiable trigger of ACLF (30%). The most common infections are spontaneous bacterial peritonitis, sepsis and pneumonia (2). Of note, during early infection stages, cirrhotic patients display higher levels of pro-inflammatory cytokines as compared to non-cirrhotic ones (13). In sepsis-induced ACLF, bacteria, and PAMPs are recognized by PRRs, which results in activation of TLR and NLR signaling pathways. In turn, this increases the activity of transcription factors involved in the production of pro-inflammatory cytokines and the activation of immune cells. TLR4 is activated in response to bacterial LPS through a recognition receptor complex that involves the co-receptors CD14 and MD-2. Downstream TLR4, LPS may activate two different circuits: MyD88-dependent or independent signaling pathways. The MyD88-dependent pathway involves nuclear translocation of NF-κB (14). This transcription factor induces the release of pro-inflammatory cytokines such as TNFα, IL-6, and IL-1β in response to infection, and, interestingly, NF-κB has also been implicated in the pathogenesis of several liver diseases, including viral hepatitis, steatohepatitis, and hepatocellular carcinoma. In contrast, the MyD88-independent pathway leads to phosphorylation of the interleukin regulatory factor three, which increases the production of type-I interferon. LPS may be also recognized in the cytoplasmic compartment by the pro-inflammatory caspases 4 and 5. Activation of NLR by caspases results in up regulation of pro-inflammatory cytokines, such as IL-1β, which are then released to recruit and activate additional inflammatory cells (15).

##### Alcohol-induced ACLF

Excessive alcohol consumption induces SI through both sterile and non-sterile mechanisms. First, alcohol alters the amount and composition of the gut microbiota. In addition, alcohol metabolism directly affects the intestinal epithelial barrier, disrupting the integrity, and altering the expression of tight junction proteins. Dysbiosis and increased intestinal permeability promote translocation of bacteria and PAMPs, which activates proinflammatory immune receptors leading to increased production of cytokines and the activation and recruitment of neutrophils to the liver (11).

In addition, sterile stimuli also contribute to inflammation. Ethanol promotes the release of mitochondrial cytochrome c and increased expression of the Fas ligand. This leads to hepatocyte apoptosis through caspase-3 dependent pathways (16). Further, oxidative stress and hepatocyte reactive oxygen species production mediate alcohol-induced liver injury, increasing the activity of cytochrome P-450 2E1 (17). The subsequent mitochondrial damage and TNF-α overproduction induce endoplasmic reticulum–dependent apoptosis and lipid synthesis up-regulation (18).

The above mechanisms are critically exacerbated in alcoholic hepatitis, which is the second most frequent identifiable trigger of ACLF (20%) (2). Extensive damage to the hepatocytes leads to cell death thus generating more DAMPs, which amplifies and perpetuates the inflammatory response.

### Immune System Cell Paralysis in ACLF

Despite exacerbated SI, patients with ACLF paradoxically feature a compromised effective immune response against pathogens which increases the risk of bacterial infection (19). This was documented in the CANONIC study in which the rate of bacterial infections increased in steps according to ACLF grade (2). An excessive compensatory anti-inflammatory response along with exhaustion and dysregulation of immune effector cells, underlie the pathogenesis of this paralysis (19). Compensatory anti-inflammatory response syndrome (CARS) has been described in patients with high grade SI due to sepsis, trauma, burns, or tissue injury (20). CARS is characterized by lymphocyte anergy and reduced numbers due to apoptosis (21), decreased cytokine production and HLA receptors upon monocyte stimulation (22), and increased expression of anti-inflammatory cytokines such as IL-10 (23). Most of these alterations have been, accordingly, described in patients with ACLF, as described below.

#### Evidence

In ACLF, the inadequate immune cell response against pathogens affects both the innate and adaptive arms of the immune system. Circulating **monocytes** in patients with ACLF show decreased HLA-DR expression, which impairs antigen presenting capacity and TNFα production in response to LPS stimulation (19). The clinical relevance of these findings was supported by a link observed between reduced monocyte HLA-DR expression and survival in critically ill cirrhotic patients (24). Additionally, **neutrophils** in alcoholic hepatitis-related ACLF show reduced phagocytic capacity and high expression levels of CXCR1/CXCR2 receptors, which contributes to hepatocyte death through early apoptosis and necrosis (25), and is associated with a greater risk of infection, organ failure and mortality. Interestingly, neutrophil dysfunction was reversed by *ex vivo* removal or neutralization of the endotoxin (26). In addition, the inefficient immune cell response of ACLF is not limited to the innate arm but extends to the T lymphocyte compartment, whose cells feature the increased expression of suppressor receptors (27).

#### Mechanisms

The exact mechanisms underlying immune cell paralysis are not fully understood, although several events contribute to its pathogenesis: (i) an excessive inhibitory immunoregulatory response triggered to counteract the massive SI, (ii) the exhaustion of effector immune system cells subjected to persistent chronic stimuli of enteric origin, and (iii), the dysfunction of immune effectors related to the metabolic and neuroendocrine abnormalities associated with hepatic insufficiency.

- As in other critical pathological scenarios such as sepsis, in ACLF, CARS responses lead to systemic deactivation of the immune system in an attempt to rescue homeostasis from an excessive inflammatory state. IL-10, primarily produced by monocytes and, to a lesser extent, lymphocytes, is the main anti-inflammatory cytokine involved in CARS (28). IL-10 modulates NF-kB activity and decreases TNFα, IL-1, IL-6, IL-8, and IL-12 secretion by monocytes (29). It also reduces the production of reactive oxygen intermediates, platelet activating factors and chemokines (28). The magnitude of CARS on hospital admission, measured by increased levels of IL-10, has been shown to predict a poor outcome in patients with ACLF (28). In murine models of liver fibrosis (bile duct ligation and CCl4), translocation of gut microbiota induces the overexpression of IFN-I in the liver and IL-10 in myeloid cells, which consequently impairs the antibacterial ability of myeloid cells (30). Monocytes from patients with ACLF display elevated frequencies of interleukin IL-10-producing cells, reduced human leucocyte antigen DR isotype expression and impaired phagocytic and oxidative burst capacity. This immunotolerant phenotype of monocytes/macrophages in ACLF may be partially restored by the metabolic reprograming of the cells using a pharmacological inhibitor of glutamine synthetase (31).
  Additionally, increased numbers of monocytes and macrophages expressing MER receptor tyrosine kinase (MERTK) have been documented in patients with ACLF. MERTK is involved in down-regulation of innate immune responses aimed at resolving inflammation (32). Activation of MERK in monocytes inhibits TLR activation and proinflammatory cytokine production. The number of MERK+ cells in ACLF has been found to correlate with disease severity and inflammation. Of note, the *ex vivo* addition of an inhibitor of MERTK, was able to rescue the production of inflammatory cytokines upon LPS stimulation (33).
- The exhaustion of immune system cells exposed to persistent antigen and inflammatory signals also occurs in other conditions such as acute and chronic infections or cancer (34). In ACLF, this exhaustion is due to chronic and exacerbated translocation of bacteria and PAMPs from a leaky gut (35), as well as increased DAMPs released from injured hepatocytes. In fact, bowel decontamination with antibiotics was observed to normalize the activation state, restore phagocytosis, and increase TNFα production upon LPS stimulation of intestinal dendritic cells in a cirrhotic rat model of bacterial translocation (35). In patients with alcoholic hepatitis-related ACLF, LPS-mediated activation of TLRs induces pronounced impairment of neutrophil function (phagocytosis, and oxidative burst), associated with the reduced production of interferon-gamma by T cells mediated by increased IL-10 production (27). In addition, T-cells from these patients express higher levels of immune inhibitory receptors (specifically, programmed cell death-1, and T-cell immunoglobulin and mucin domain). Blockade of these receptors with specific antibodies was able to restore the antimicrobial activities of neutrophils and T cells (27). Of note, all these events take place in a T-cell compartment that is already retracted in cirrhosis due to defective thymopoiesis and increased activation-driven cell death (36). Monocyte oxidative burst in alcoholic hepatitis is also impaired due to reduced NADPH oxidase expression. A defective oxidative burst has been shown to predict the development of infections and death in this context (37).
- Finally, metabolic and neuroendocrine abnormalities subsequent to severe liver insufficiency can impair immune cell functions. In this regard, prostaglandin E2, which appears elevated in patients with acute decompensation of cirrhosis (38), contributes to monocyte dysfunction by inhibiting NADPH oxidase-mediated bacterial killing (39). Prostaglandin E2 induced immunosuppression was antagonized by indomethacin and by the binding effect of albumin. Accordingly, decreased levels of serum albumin (below 30 mg/ml) predicted susceptibility to infection (38). Other distinctive metabolic features of liver failure, such as hyperammonemia and hyponatremia, contribute to the immune dysfunction of cirrhosis. Hyponatremia acts synergistically with ammonia to cause neutrophil swelling and impaired phagocytosis, an effect abrogated by p38 mitogen-activated protein kinase signaling inhibition (40). SI also activates tryptophan metabolism increasing its degradation via the kynurenine pathway. Under physiological conditions, kynurenine metabolites are synthesized in the liver by local enzymes, however, in the setting of SI and hepatic insufficiency, there is a predominant extrahepatic metabolism. Kynurenine pathway activity is markedly increased in cirrhotic patients with ACLF, and a high baseline activation independently predicts mortality in this situation (41). In fact, in ACLF, kynurenine metabolites seem to contribute to systemic circulatory dysfunction by increasing nitric oxide production and oxidative stress, immunosuppression by increasing IL-10 release, and brain dysfunction.

## Mechanisms of Organ Failure in ACLF

**Figure 2:**
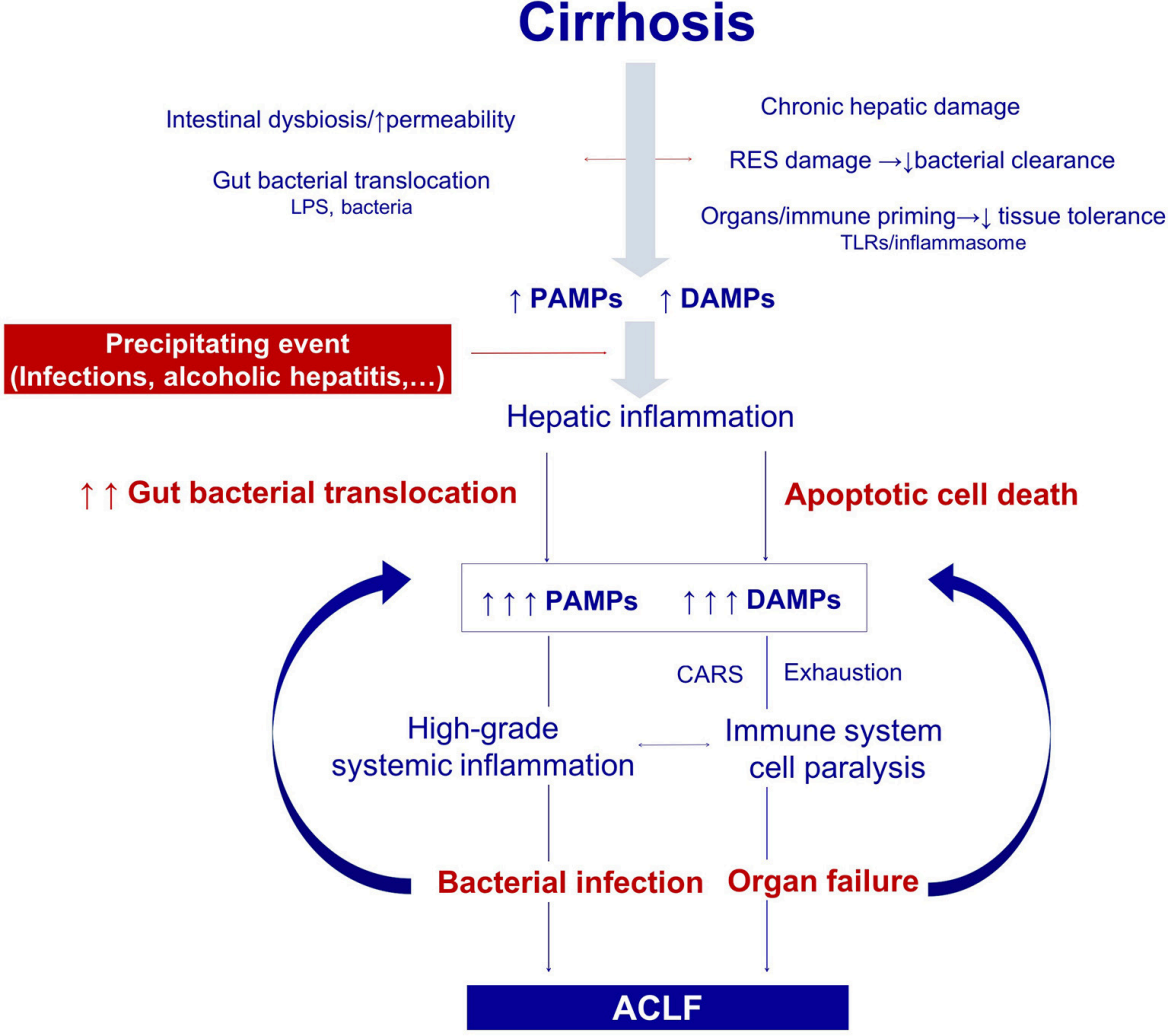
Pathogenesis of the organ failure in ACLF. Architectural changes of the liver due to cirrhosis, derange the surveillance role of the hepatic reticuloendothelial system (RES), and contribute to impaired endotoxin and bacterial clearance. The progressive damage to the hepatocytes impairs the synthesis of proteins involved in the innate immune response. In addition, gut dysbiosis and increased permeability exacerbate bacterial translocation and priming of the immune system cells leading to low-grade systemic inflammation. Monocytes and macrophages persistently exposed to low levels of endotoxin eventually become unresponsive to further endotoxin challenge. Additionally, the loss of the protective-tissue intrinsic mechanisms of tolerance in the cirrhotic liver results in a severe outcome after any noxa. In this scenario, sterile or infectious acute events induce hepatic inflammation and necrosis, and thus generate more PAMPs and DAMPs. Activation of pattern recognition receptors by increased PAMPs and DAMPs triggers a massive release of cytokines and hepatocyte death by immune mediated apoptosis and necrosis. Organ failure is not only related to the collateral effects of the immune response, but also to the hemodynamic derangement present in ACLF. In parallel, an excessive compensatory anti-inflammatory response trying to counteract the high-grade systemic inflammation, and the exhaustion and dysfunction of key innate and adaptive immune system cells, lead to a functional immune cell paralysis, which increases the risk of infections. Therapies targeting the elements of the altered immune response or the factors leading to it are promising in ACLF, including those aiming to correct gut dysbiosis and reduce bacterial translocation, to reduce high-grade systemic inflammation, and to improve the immune cell dysfunction. RES, Reticuloendothelial system; DAMPs, damage associated molecular patterns; PAMPs, pathogen associated molecular patterns; LPS, Lipopolysaccharides; PRRs, pattern recognition receptors; TLR-4, Toll like receptor 4; NFkB, nuclear factor kappa-light-chain-enhancer of activated B cells; CAR, compensatory anti-inflammatory response.

ACLF is defined by the presence of hepatic and/or extrahepatic organ failure, which differentiates this syndrome from acute decompensation of cirrhosis. Despite early detection and intervention on the precipitant events, the dysfunctional immune response leads to further organ failure, which is mainly driven by the massive systemic inflammatory response (Figure 2).

A. **General mechanisms of organ failure in high-grade SI**

The mechanisms underlying organ failure are related not only to the hemodynamic derangement, but also to cell dysfunction and cell death (apoptosis and necrosis) induced by the excessive inflammatory response (42). The term “immunopathology” refers to the collateral damage of the activated immune cells and derived factors on other cells and tissues resulting in organ failure. The degree of immunopathology correlates with the magnitude, duration, and type of the immune response. In this regard, effector responses including IFNγ-activated macrophages, recruited neutrophils, cytotoxic lymphocytes or T helper 17 cells are related to a high immune-mediated tissue damage and consequently to an increased risk of organ failure (43). In ACLF, significantly higher levels of cytokines such as IL-6 or IL-8, among others, are found (4). IL-6 is critical for lymphocytes T helper 17 responses, which contribute to neutrophil production and activation. On the other hand, IL-8 induces neutrophil chemotaxis and phagocytosis. Recruited neutrophils release reactive oxygen species and proteolytic enzymes contributing to immunopathology and cell death.

In addition, it has been suggested that cirrhosis is associated with a decreased capacity of tolerance to infections (44). Tolerance aims to reduce the negative impact of infection on host fitness, decreasing the susceptibility to tissue damage caused by the pathogens, and the immune response (45). In fact, cirrhotic livers are abnormally sensitive to *in vivo* LPS-induced TNFα-mediated apoptosis, due to an altered unfolded protein response, which might contribute to liver damage under bacterial challenge in cirrhosis (44). In agreement with the latter experimental findings, the lack of tolerance seems to be more marked in those patients with ACLF without previous acute decompensations (2), and correlates with an increased mortality in this group.

Tissue hypoperfusion and hypoxia secondary to microthrombi formation, blood maldistribution, tissue edema, and decreased perfusion pressure as a consequence of nitric oxide overproduction, are key to the pathogenesis of organ failure. Excessive nitric oxide also impairs mitochondrial function leading to altered cell oxygen consumption and ATP synthesis. Neutrophil recruitment to injured tissues may have a detrimental effect through the production of lysosomal enzymes and superoxide free radicals (46). Finally, inflammation promotes tissue factor release, which in turn triggers the extrinsic coagulation cascade and production of activated thrombin leading to microthrombi formation and eventually disseminated intravascular coagulation.

B. **Specific mechanisms of organ failure in ACLF**

Portal hypertension in cirrhosis induces profound hemodynamic derangement characterized by splanchnic arterial vasodilation and hyperdynamic circulation with high cardiac output and low systemic vascular resistance. In ACLF, cytokine-induced overproduction of nitric oxide and reactive oxygen species intensifies arterial vasodilation, decreases mean arterial pressure and impairs left ventricular function, contributing to the hemodynamic deterioration (47).

Reduced arterial pressure and activation of neurohumoral compensatory mechanisms lead to renal arterial vasoconstriction and prompt kidney hypoperfusion. Hemodynamic derangement, but also ischemic acute tubular necrosis due to intense renal capillary leukocyte infiltration, microthrombosis, cell apoptosis, and mitochondrial injury contribute to acute kidney failure in ACLF (48). The relevance of mitochondrial dysfunction in the pathogenesis of sepsis-induced renal failure is increasingly recognized. During sepsis, several mitochondrial functions, key for cellular homeostasis, are altered. In particular, oxidative phosphorylation and ATP production decrease, while reactive oxygen species production and apoptosis augment (49). Affected mitochondria also release DAMPs, such as mitochondrial DNA, which further contribute to amplify the immune response (50). The involvement of inflammatory mechanisms rather than systemic circulatory dysfunction in renal failure in ACLF explains the lower efficacy of terlipressin and albumin in this context as compared to decompensated cirrhosis (51).

Hepatocytes are protected from TNFα-induced apoptosis through activation of NF-kβ-dependent anti-apoptotic mechanisms (52). However, in cirrhotic rats, hepatocyte endoplasmic reticulum stress prevents the translation of these antiapoptotic messenger RNAs into proteins (44). Under these circumstances of failure of protective tissue-intrinsic mechanisms, it is likely that the response of the liver to a precipitant event can be particularly severe. This contributes to liver injury and failure upon LPS-mediated production of TNFα. Additionally, LPS engagement to PRRs is related to hepatocyte necrosis and neutrophil infiltration induced by endothelin 1. Treatment with a non-selective endothelin receptor antagonist has been observed to decrease intrahepatic neutrophil infiltration and increase *in vivo* survival in endotoxin-challenged cirrhotic rats (53).

Collectively, these lines of evidence support the immunopathological mechanisms underlying organ failure in cirrhosis and ACLF.

## Therapeutic Strategies Targeting the Immune System in ACLF

Currently, the treatment of ACLF is based on organ support and correction of the precipitating events when possible. However, the potential reversibility of the immune alterations, at least in *ex vivo* models, suggests promising therapeutic targets for patients with ACLF.

### Therapies Targeting the Gut

Therapies targeting gut microbiota may potentially improve hemodynamic derangement and immune cell dysfunction in ACLF. Intestinal decontamination with non-absorbable antibiotics has been shown to ameliorate systemic vascular nitric oxide production, inflammation, and hemodynamic alterations in experimental models and in human cirrhosis (35, 54–57). As described above, bowel decontamination was also able to normalize dendritic cell dysfunction and increase TNF-α production in experimental cirrhosis (35). Currently, clinical trials are further investigating the efficacy of simvastatin and rifaximin in patients with decompensated cirrhosis to prevent ACLF, reduce complications and hospital readmissions, and improve cost-effectiveness, quality-of-life and survival (LIVERHOPE project, EU H2020).

A different approach targeting the gut involves the use of poorly absorbable, adsorptive materials, such as synthetic adsorptive nanoporous carbons that bind gut-derived toxins and bacterial products. A novel synthetic activated carbon (Yaq-001) has shown promising results in rats with biliary cirrhosis (58). Clinical trials using Yaq-001 are ongoing as part of the European Commission Horizon 2020 program (carbalive.eu).

### Therapies Targeting High-Grade Inflammation

Preclinical studies have shown that treatment with the IL1 receptor antagonist anakinra reduces liver inflammation and neutrophil infiltration, improving hepatocyte regeneration, and recovery in mouse models of ACLF (59). Conversely, IL-22 has been shown to protect and repair liver injury in mice with alcoholic hepatitis, targeting signal transducer, and activator of transcription 3 (60). The antiapoptotic, proliferative, and antimicrobial effects of IL-22 are potentially beneficial for patients with ACLF. The safety and efficacy of this molecule is currently being tested in patients with alcoholic hepatitis (ClinicalTrials.gov ID: NCT02655510).

Targeting inflammation and apoptosis mediated by caspases has yielded promising results in chronic hepatitis C and non-alcoholic fatty liver disease (61, 62). In ACLF, the role of pan-caspase inhibitors (such as Emricasan) has also been explored. Unfortunately, clinical benefits assessed by severity of liver disease (MELD or CLIF-C ACLF score) or mortality, has not been demonstrated in this context so far (63).

### Therapies Targeting the Immune Cell Paralysis

The *in vitro* rescue of cell function using immune targeted strategies, and recapitulation of immune paralysis using serum from ACLF patients, suggest that cell reprograming leading to immunodeficiency may be reversible, and provide evidence for new therapeutic targets.

Albumin has not only shown hemodynamic effects as a plasma expander but has also proven immune functions. The latter arise from its capacity to bind and inactivate pro-inflammatory molecules such as LPS and other bacterial products, reactive oxygen and nitrogen species, and prostaglandins (64), thus supporting its potential therapeutic effects in patients with ACLF (65).

Granulocyte colony-stimulating factor (G-CSF) promotes mobilization of bone marrow stem cells, which has been shown to promote hepatic regeneration and restore neutrophil function (66). In patients with ACLF, treatment with G-CSF increased the number of CD34(+) stem cells, and was significantly associated with higher survival rates (67, 68). Currently, several clinical trials are underway to confirm G-CSF efficacy and safety in this context (ClinicalTrials.gov ID: NCT01383460, NCT02788240, NCT03162419, and NCT02669680). Interestingly, it has been suggested that liver-derived mesenchymal stem cells produce higher levels of pro-angiogenic, anti-inflammatory, and anti-apoptotic cytokines than stem cells derived from the bone marrow (69). The safety and efficacy of these cells in ACLF are currently under investigation (ClinicalTrials.gov ID: NCT02946554).

## Conclusion

Massive SI and immune cell paralysis associated with ACLF represent the extreme severity of CAID in response to an infectious or sterile challenge. The severe immune disturbance plays a pivotal role in the pathogenesis of the distinctive features of ACLF: organ failure and bacterial infection susceptibility. Excessive SI in ACLF results from the massive activation and dysfunction of an innate immune system challenged by increased PAMPs and DAMPs. SI leads to cell and tissue immunopathology contributing to hepatic and extrahepatic organ failure. Concomitantly, the course of ACLF is associated with a disproportionate compensatory anti-inflammatory response along with exhaustion and dysregulation of the innate and adaptive arms of the immune system, with the subsequent functional immune paralysis that confers a higher risk of infections and further ACLF progression. Therapies aimed at preventing the development of these immune disturbances in ACLF are novel promising strategies to improve survival in these patients.

## Author Contributions

The manuscript was drafted by RM-M and AA. MA-M and AA critically reviewed the manuscript. All the authors approved the final draft for submission. The guarantor of the manuscript is AA.

### Conflict of Interest Statement

The authors declare that the research was conducted in the absence of any commercial or financial relationships that could be construed as a potential conflict of interest.
